# Zinc Oxide Nanoparticles Blunt Potassium-Bromate-Induced Renal Toxicity by Reinforcing the Redox System

**DOI:** 10.3390/molecules28135084

**Published:** 2023-06-29

**Authors:** Ibrahim M. Alhazza, Iftekhar Hassan, Hossam Ebaid, Jameel Al-Tamimi, Zafrul Hasan

**Affiliations:** 1 Department of Zoology, College of Science, King Saud University, Riyadh 11451, Saudi Arabia; ihazza@ksu.edu.sa (I.M.A.); hossamebaid1969@gmail.com (H.E.); jhattamimi@gmail.com (J.A.-T.); 2 College of Nursing, King Saud University, Riyadh 11451, Saudi Arabia; zhasan@ksu.edu.sa

**Keywords:** zinc oxide, nanoparticles, potassium bromate, nephrotoxicity, oxidative stress

## Abstract

Potassium bromate (PB) is a general food additive, a significant by-product during water disinfection, and a carcinogen (Class II B). The compound emits toxicity depending on the extent of its exposure and dose through consumable items. The current study targeted disclosing the ameliorative efficacy of zinc oxide nanoparticles (ZnO NPs) prepared by green technology in PB-exposed Swiss albino rats. The rats were separated into six treatment groups: control without any treatment (Group I), PB alone (Group II), ZnO alone (Group III), ZnO NP alone (Group IV), PB + ZnO (Group V), and PB + ZnO NPs (Group VI). The blood and kidney samples were retrieved from the animals after following the treatment plan and kept at −20 °C until further analysis. Contrary to the control (Group I), PB-treated rats (Group II) exhibited a prominent trend in alteration in the established kidney function markers and disturbed redox status. Further, the analysis of the tissue and nuclear DNA also reinforced the biochemical results of the same treatment group. Hitherto, Groups III and IV also showed moderate toxic insults. However, Group VI showed a significant improvement from the PB-induced toxic insults compared to Group II. Hence, the present study revealed the significant therapeutic potential of the NPs against PB-induced nephrotoxicity in vivo, pleading for their usage in medicines having nephrotoxicity as a side effect or in enhancing the safety of the industrial use of PB.

## 1. Introduction

Potassium bromate (PB) has been widely used as a food additive and a processing and maturing agent at the industrial level. It has been used in many consumer items such as beverages, cosmetics, food packaging, mouth hygiene products, and pharmaceuticals since its discovery in 1900 [1,2]. During processing with the compound in these industries, traces of it get into the living system. Besides their regular usage in bakery products, many packaged beverages and municipality-treated tap water post ozonization during its filtration process are two significant sources of human exposure [3]. Internalizing the compound in a living system, PB generates free radicals during its biotransformation, resulting in the perturbation of cellular redox status and the associated tissue damage and macromolecular oxidative breakdown. It has been documented that the metabolically processed PB can produce even more aggressive products (bromate and bromide radicals) besides ROSs, which can demolish various cellular components and critical cellular structures such as mitochondria, Golgi bodies, and the nucleus [4,5,6]. Further, after getting into the nucleus, these aggressive products can cause chromosomal aberration and oxidative damage to nucleic acids and vital proteins to a greater extent [7]. This chemical has been classified as a Class 2 B carcinogen due to its strong oxidant and mutagenic properties in vivo [7]. Hence, continuous exposure to it can cause many pathological conditions, including renal failure, cirrhosis, and even cancer [8,9].

Currently, nanotechnology is one of the most-advancing sciences for improving medical, environmental, and engineering consumer items. Much literature has highlighted its potential to enhance the treatment modalities of numerous complex diseases and add quality to the patient’s life [10,11,12]. Nanoparticles have been in the spotlight for contemporary researchers exploring their usage in the diagnosis of diseases, novel strategies in drug delivery, or enhancing the efficacy of drugs, besides being used as an enhancer in the food industry, cosmetics, varnishes, electronic devices, sports items, environmental restoration, and packaging [13,14]. ZnO nanoparticles are among the most-extensively researched and applied nanoparticles since the discovery of nanotechnology. The unique physicochemical features (surface charge, size, and shape) of some of the metallic nanoparticles (NPs), such as zinc oxide nanoparticles (ZnO-NPs), make them suitable candidates to be used in the consumer industries of cosmetics, paints, electronic devices, catalysis, energy, bioremediation, food technology, biotechnology, and medicine [15,16,17]. Over three-hundred types of biological functions are directly or indirectly linked to this particular element, Zn being a component in enzymes, proteins, and transcription factors contributing to cell signaling, immunity, structural integrity, and cellular proliferation and differentiation [18]. With the increasing usage of NPs, the assessment of their safety to humankind is paramount in the latest research facets [19]. They enter the living system as a nanostructured ingredient in numerous human fields through different routes such as ingestion, respiration, or skin penetration [20]. Many investigators have reported that, once NPs enter the living system through any portal of entry, including oral administration, they can be absorbed and distributed by the systemic circulation to various tissues, including the brain [21]. Zinc oxide nanoparticles (ZnO-NPs) are among the most-commonly used NPs among various metal oxides because of their ease in synthesis, bio-compatible physico-chemical properties, and wide range of utilization in dyes, toothpaste, cosmetics, textiles, medicines, wall paints, and other building materials [20,22]. Many recent studies have indicated that NPs have excellent antimicrobial, antifungal, and anticancer activities [23,24]. With greater acceptability and a low toxicity profile in vivo, they are very propitious in the horizon of pharmaceuticals as an adjuvant to established drugs and a means of drug delivery [21,25,26]. In addition, many investigators have demonstrated these NPs to be pharmacologically active at moderate doses with excellent efficacy [27,28].

As both PB and NPs are filtered by the kidney after biotransformation by the liver, toxicity to the kidneys is a significant health concern. The present work was dedicated to evaluating the efficacy of NPs in ameliorating PB-induced renal toxicity. The current result was based on the interaction between the two compounds in vivo leading to NPs’ blunting of the PB-mediated renal toxicity by orchestrating oxidative stress parameters and biochemical factors. Furthermore, these results were established by histopathology and comet assay in the kidney samples of the treated rodents.

## 2. Results

### 2.1. Effect on Kidney Function Markers

#### 2.1.1. Urea

**Figure 1:**
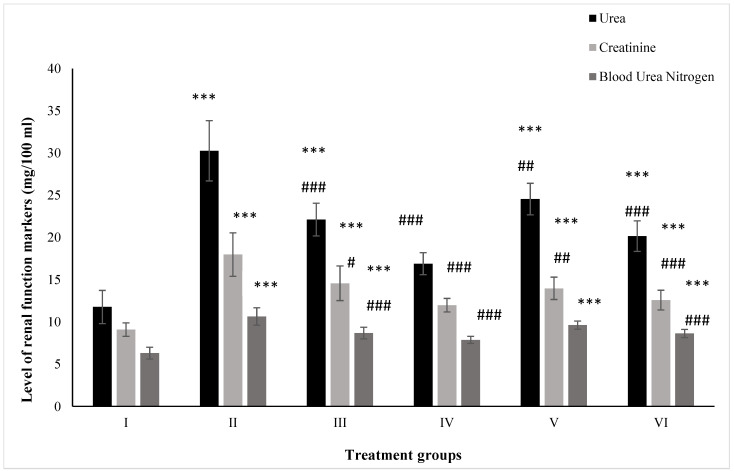
All the data are expressed as the mean ± the SD for urea, creatinine, and BUN in serum samples of the rats from: Group I (control); Group II (single dose of PB at 100 mg/kg); Group III (multiple doses of ZnO at 5 mg/kg); Group IV (multiple doses of ZnO-NP at 5 mg/kg); Group V (single dose of PB at 100 mg/kg with multiple doses of ZnO at 5 mg/kg; Group VI (single dose of PB at 100 mg/kg with multiple doses of ZnO-NPs at 5 mg/kg). The asterisk marks *** indicate statistically significant from the control (Group I) at *p* ≤ 0.05, *p* ≤ 0.01, and *p* ≤ 0.001, respectively, while #, ##, and ### indicate statistically significant from Group II at *p* ≤ 0.05, *p* ≤ 0.01, and *p* ≤ 0.001, respectively (*n* = 5–6).

Urea is one of the primary renal markers for assessing kidney function in vivo. Group II (PB-treated) demonstrated an increase in its level by 157.31% as compared to the control, Group I (control). ZnO- and ZnO-NP-treated Groups III and IV showed a rise in their level by 88.01% and 43.62%, respectively, compared to the control. However, the administration of ZnO and ZnO-NPs in the groups pretreated with PB, Groups V and VI, exhibited elevated levels by 18.90% and 33.41% compared to the positive control, Group II (Figure 1).

#### 2.1.2. Creatinine

Creatinine is also a crucial renal marker for the assessment of renal function. Group II displayed a rise in its level by 97.90% after treatment with PB with respect to the control, Group I, while ZnO- and ZnO-NP-treated Groups III and IV exhibited enhanced levels by 60.46% and 31.82% compared to the control. Hitherto, PB-pretreated Groups V and VI showed a rise in their level by 22.25% and 30.05% after dosing with ZnO and ZnO-NPs compared to Group II (Figure 1).

#### 2.1.3. BUN

The mice from Groups II, III, and IV showed an increase in level by 68.73%, 36.77%, and 24.76% after treatment with PB, ZnO, and ZnO-NPs with respect to Group I, while Groups V (PB+ZnO) and VI (PB+ZnO-NPs) exhibited increased levels by 9.59% and 18.90% as compared to the positive control, Group II (Figure 1).

### 2.2. Effect on Lipid Profile

#### 2.2.1. LDL

**Figure 2:**
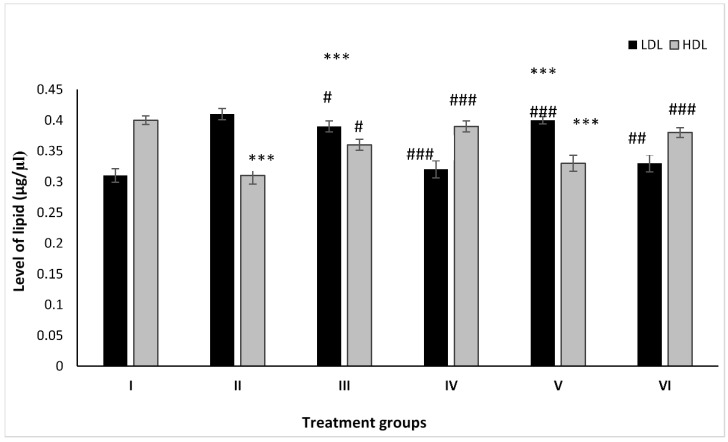
All the data are expressed as the mean ± the SD for LDL and HDL in serum samples of the rats from: Group I (control); Group II (single dose of PB at 100 mg/kg); Group III (multiple doses of ZnO at 5 mg/kg); Group IV (multiple doses of ZnO-NP at 5 mg/kg); Group V (single dose of PB at 100 mg/kg with multiple doses of ZnO at 5 mg/kg; Group VI (a single dose of PB at 100 mg/kg with multiple doses of ZnO-NPs at 5 mg/kg). The asterisk marks *** indicate statistically significant from the control (Group I) at *p* ≤ 0.05, *p* ≤ 0.01, and *p* ≤ 0.001, respectively, while #, ##, and ### indicate statistically significant from Group II at *p* ≤ 0.05, *p* ≤ 0.01, and *p* ≤ 0.001, respectively (*n* = 5–6).

Groups II, III, and IV showed an increase by 32.25%, 25.80%, and 3.22% after treatment with PB, ZnO, and ZnO-NPs with respect to Group I, whereas Groups V and VI pre-treated with PB demonstrated a decrease in its level by 2.43% and 19.51% after administration with ZnO and ZnO-NPs as compared to Group II (Figure 2).

#### 2.2.2. HDL

The animal Groups II, III, and IV demonstrated dip in its level by 22.25%, 9.75%, and 2.50% after treatment with PB, ZnO, and ZnO-NPs with respect to Group I, while the combination-treated Groups V and VI exhibited an increase in its level by 6.43% and 23.15% as compared to Group II (Figure 2).

### 2.3. Effect on Kidney Toxicity Markers

#### 2.3.1. Albumin

**Figure 3:**
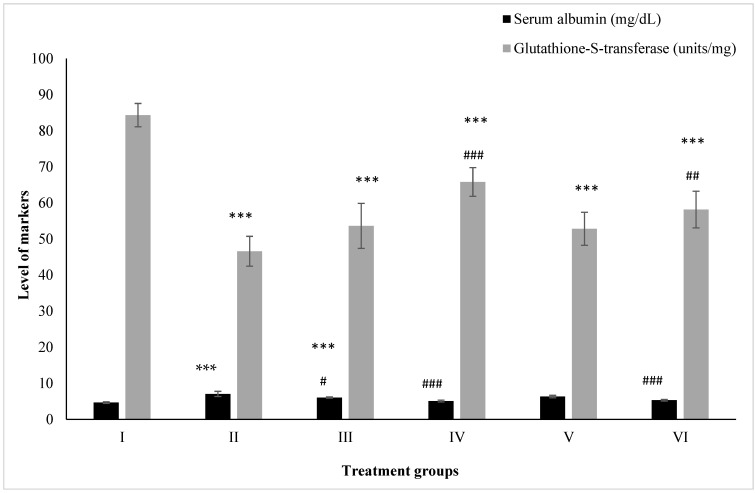
All the data are expressed as the mean ± the SD for albumin and GST in serum samples of the rats from: Group I (control); Group II (single dose of PB at 100 mg/kg); Group III (multiple doses of ZnO at 5 mg/kg); Group IV (multiple doses of ZnO-NP at 5 mg/kg); Group V (single dose of PB at 100 mg/kg with multiple doses of ZnO at 5 mg/kg; Group VI (single dose of PB at 100 mg/kg with multiple doses of ZnO-NPs at 5 mg/kg). The asterisk marks *** indicate statistically significant from the control (Group I) at *p* ≤ 0.05, *p* ≤ 0.01, and *p* ≤ 0.001, respectively, while #, ## and ### indicate statistically significant from Group II at *p* ≤ 0.05, *p* ≤ 0.01, and *p* ≤ 0.001, respectively (*n* = 5–6).

Group II showed an elevation in its level by 50.74% compared to the control, Group I, while Groups III and IV showed an increase of 29.42% and 8.74%. However, Groups V and VI demonstrated a decline in its level by 10.32% and 24.61% compared to Group II (Figure 3).

#### 2.3.2. Glutathione-S-Transferase

Groups II, III, and IV administered PB, ZnO, and ZnO-NPs displayed a decrease in its level by 44.74%, 36.40%, and 21.96% with respect to the control (Group I), while Groups V and VI treated with the combinations of PB with ZnO and ZnO-NPs demonstrated an increase in its level by 13.35% and 24.79% with respect to Group II (Figure 3).

### 2.4. Effect on Antioxidant Parameters

#### 2.4.1. CAT

**Figure 4:**
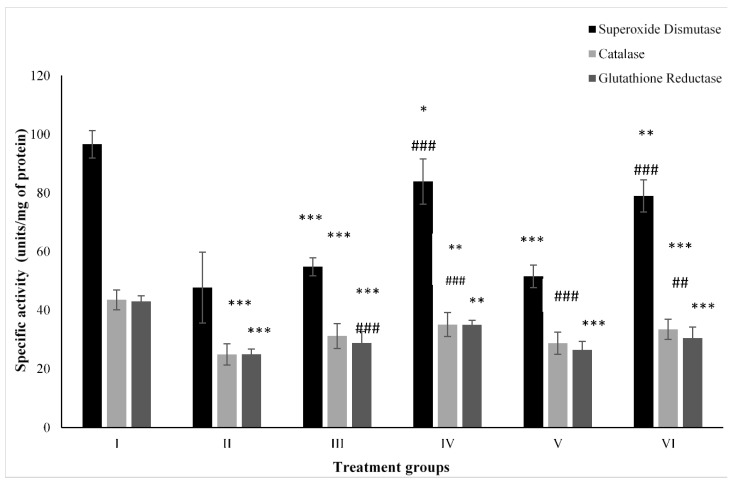
All the data are expressed as the mean ± the SD to show the activity of superoxide dismutase (SOD), catalase (CAT), and glutathione reductase (GR) in tissue homogenates of the rats from: Group I (control); Group II (single dose of PB at 100 mg/kg); Group III (multiple doses of ZnO at 5 mg/kg); Group IV (multiple doses of ZnO-NP at 5 mg/kg); Group V (single dose of PB at 100 mg/kg with multiple doses of ZnO at 5 mg/kg; Group VI (single dose of PB at 100 mg/kg with multiple doses of ZnO-NPs at 5 mg/kg). The asterisk marks *, **, and *** indicate statistically significant from the control (Group I) at *p* ≤ 0.05, *p* ≤ 0.01, and *p* ≤ 0.001, respectively, while ##, and ### indicate statistically significant from Group II at *p* ≤ 0.05, *p* ≤ 0.01, and *p* ≤ 0.001, respectively (*n* = 5–6).

Figure 4 shows the activity of CAT. Group II showed a compromised level of CAT by 42.89% in comparison to the control followed by Groups III and IV. However, the combination Groups V and VI showed enhancement in its activity by 15.34% and 34.58% compared to Group II (Figure 4).

#### 2.4.2. SOD

Group II after PB treatment, showed depletion in its activity by 50.61% compared to the control, Group I, while ZnO-treated Group III and ZnO-NP-treated Group IV showed a dip by 43.25% and 13.15%, respectively. However, the combination-treated Groups V and VI had increased activity of it by 8.05% and 65.52% compared to Group II (Figure 4).

#### 2.4.3. GR

Group II dosed with PB displayed a compromise in its level by 42% with respect to the control (Group I) followed by ZnO-treated Group III and ZnO-NP-treated Group IV. However, Group V and VI treated with the combinations of PB with ZnO and ZnO-NPs had enhanced activity by 5.94% and 21.97% compared to Group II (Figure 4).

#### 2.4.4. GSH

**Figure 5:**
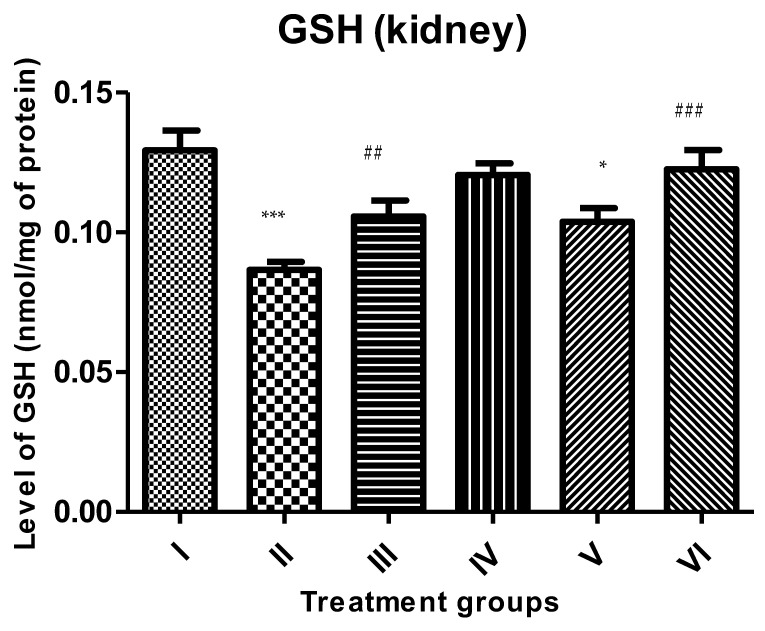
All the data are expressed as the mean ± the SD to show the level of rescued glutathione (GSH) in the tissue homogenate of the rats from: Group I (control); Group II (single dose of PB at 100 mg/kg); Group III (multiple doses of ZnO at 5 mg/kg); Group IV (multiple doses of ZnO-NP at 5 mg/kg); Group V (single dose of PB at 100 mg/kg with multiple doses of ZnO at 5 mg/kg; Group VI (single dose of PB at 100 mg/kg with multiple doses of ZnO-NPs at 5 mg/kg). The asterisk marks * and *** indicate statistically significant from the control (Group I) at *p* ≤ 0.05, *p* ≤ 0.01, and *p* ≤ 0.001, respectively, while ##, and ### indicate statistically significant from Group II at *p* ≤ 0.05, *p* ≤ 0.01, and *p* ≤ 0.001, respectively (*n* = 5–6).

A decline of 33.33% was observed in Group II in the GSH level in comparison to the control, followed by Groups III and IV. However, the treatment with the combinations of PB with ZnO and ZnO-NPs caused its replenishment by 19.76% and 41.86% compared to Group II (Figure 5).

### 2.5. Effect on Macromolecular Oxidation

#### 2.5.1. Carbonyl Content

**Figure 6:**
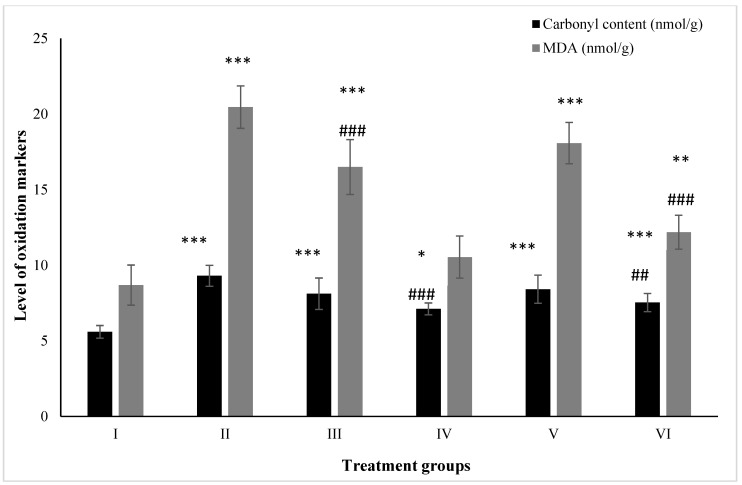
All the data are expressed as the mean ± the SD for the level of carbonyl content and total malondialdehyde in tissue homogenate of the rats from: Group I (control); Group II (single dose of PB at 100 mg/kg); Group III (multiple doses of ZnO at 5 mg/kg); Group IV (multiple doses of ZnO-NP at 5 mg/kg); Group V (single dose of PB at 100 mg/kg with multiple doses of ZnO at 5 mg/kg; Group VI (single dose of PB at 100 mg/kg with multiple doses of ZnO-NPs at 5 mg/kg). The asterisk marks *, **, and *** indicate statistically significant from the control (Group I) at *p* ≤ 0.05, *p* ≤ 0.01, and *p* ≤ 0.001, respectively, while ##, and ### indicate statistically significant from Group II at *p* ≤ 0.05, *p* ≤ 0.01, and *p* ≤ 0.001, respectively (*n* = 5–6).

The carbonyl content was enhanced by 66.36% in Group II followed by Groups III and IV at 45.08% and 27.19% compared to the control. However, Groups V and VI treated with ZnO and ZnO-NPs demonstrated a decrease in its level by 9.56% and 19.03% in comparison to Group II, respectively (Figure 6).

#### 2.5.2. MDA

MDA is one of the most-reliable biochemical markers for the assessment of lipid peroxidation. The level of MDA was found to have increased by 135.71% in Group II followed by 90.09% and 21.42% in Groups III and IV, respectively. However, Groups V and VI showed a declination in its level by 11.63% and 40.42% compared to Group II (Figure 6).

### 2.6. Effect on Nuclear DNA

**Figure 7:**
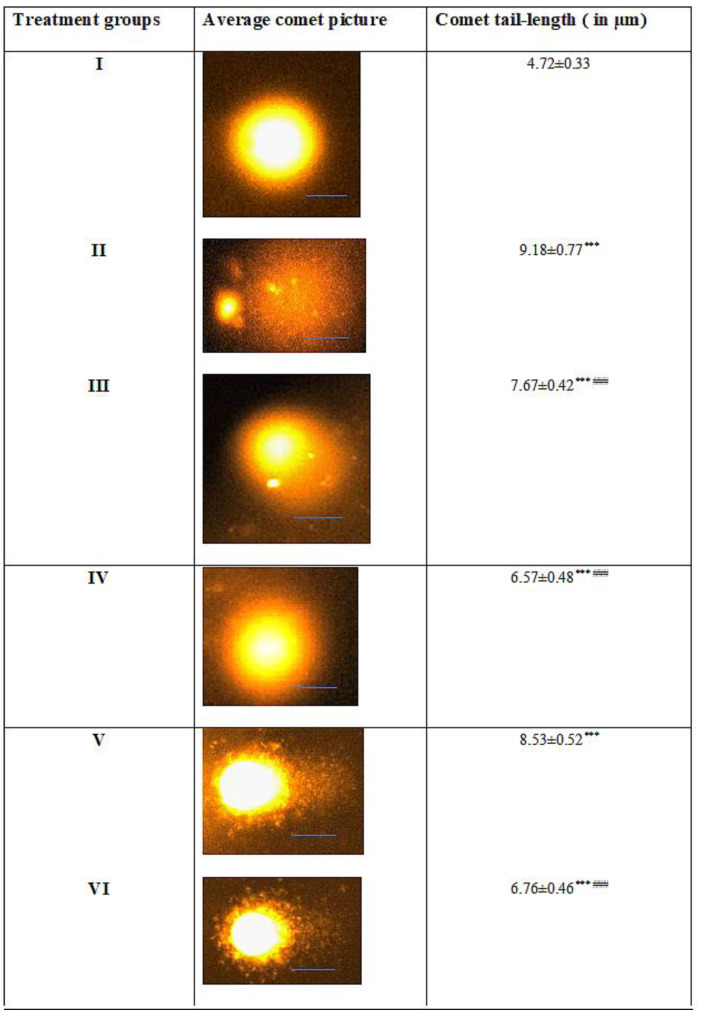
All the data are expressed as the mean ± the SD for the tail-length of the nuclear DNA of kidney cells in rats from: Group I (control); Group II (single dose of PB at 100 mg/kg); Group III (multiple doses of ZnO at 5 mg/kg); Group IV (multiple doses of ZnO-NP at 5 mg/kg); Group V (single dose of PB at 100 mg/kg with multiple doses of ZnO at 5 mg/kg; Group VI (single dose of PB at 100 mg/kg with multiple doses of ZnO-NPs at 5 mg/kg) along with a scale bar of 10 µM in each group’s average picture. The asterisk marks *** indicate statistically significant from the control (Group I) at *p* ≤ 0.05, *p* ≤ 0.01, and *p* ≤ 0.001, respectively, while ### indicate statistically significant from Group II at *p* ≤ 0.05, *p* ≤ 0.01, and *p* ≤ 0.001, respectively (*n* = 5–6). A standard scale bar (—) of 10 µm have been given in each figure.

The comet assay was conducted to assess the effect of the treatment on the integrity of the nuclear DNA of the target cells (kidney). Group II showed an increase in the tail-length by 94.49% with respect to the control, exhibiting extensive damage to the nuclear DNA. Groups III and IV also demonstrated mild damage, evidenced by an increase in their tail-lengths by 62.5% and 39.19%, respectively. However, the combination Groups V and VI exhibited restoration of the nuclear DNA as there was a decline in the tail-length by 7.08% and 26.36% in comparison to Group II (Figure 7).

### 2.7. Histological Evaluation of Kidney Samples

**Figure 8:**
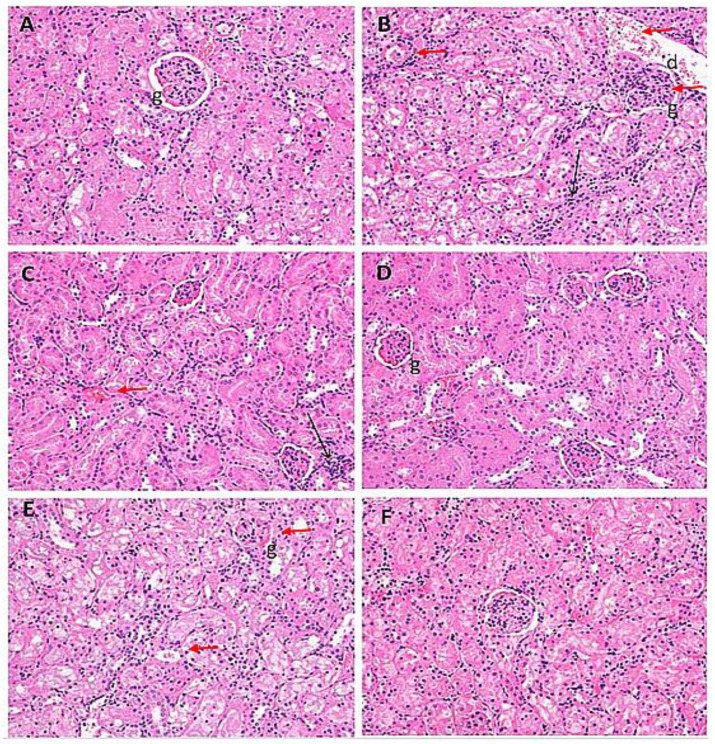
Photomicrographs of kidney cells from the treated rat groups stained with H& E snapped at 400× in serum samples of the rats from: (**A**) Group I (control); (**B**) Group II (single dose of PB at 100 mg/kg); (**C**) Group III (multiple doses of ZnO at 5 mg/kg); (**D**) Group IV (multiple doses of ZnO-NP at 5 mg/kg); (**E**) Group V (single dose of PB at 100 mg/kg with multiple doses of ZnO at 5 mg/kg; (**F**) Group VI (single dose of PB at 100 mg/kg with multiple doses of ZnO-NPs at 5 mg/kg). Black and red arrows point toward the major histological alterations in the tissue samples. In the figures, ‘d’ is for distal covulated tubules; ‘b’ for Bowman’s capsule; ‘g’ for glomeruli.

The normal renal tissues of the rats (Group I) are presented in Figure 8A. Histopathological examination of the PB-treated rats’ renal tissues (Group II) showed intensive hemorrhage in the blood vessels, which appeared dilated with some lumens resembling hyaline (indicated by arrows in Figure 8B). Sections from the same group also showed narrow urinary spaces, which may be due to edema (indicated by the black arrow in Figure 8). In addition, some blood vessels were detected inside the glomerulus with obviously disturbed cells in the collecting duct wall (Table 1). On the other hand, an overall improvement in the renal architecture was observed after treatment with ZnO nanoparticles (Group VI) in PB-challenged rats with many tissue damage markers being recovered (Figure 8D,F). Nonetheless, after the nanoparticle treatment, some glomeruli still appeared shrunken with detectable hemorrhage (Figure 8F). Histological scores confirmed an improvement after treatment with ZnO nanoparticles in Group VI (Table 1).

## 3. Discussion

Despite being an established health hazard compound and potential Group 2B carcinogen (International Agency for Research on Cancer (IARC), PB is being used in various everyday consumer items such as bakery products, packaged food products, cosmetic items, and even drinking water [5,8,29,30]. Many investigators have advocated the co-administration of natural compounds and synthetic agents with therapeutic properties to cease PB’s toxic insults, allowing its usage with null or fewer health hazards [4,31]. The current study attempted to alleviate specifically PB-induced nephrotoxicity in vivo. Intriguingly, the present investigation demonstrated that the customized administration of ZnO-NPs with the defined treatment strategy can optimize the redox status and renal markers in PB-challenged rats. The additional histological and nuclear DNA analysis (comet assay) consolidated the current results.

The present work showed how PB exerts its toxic insults in the treated animals, evidenced by significantly elevated renal function markers (urea, creatinine, and BUN) and toxicity markers (serum albumin and GST). The compound, upon long exposure, accumulates in the vital organs, including the kidneys, which damages the organs, leading to nephrotoxicity. The damage caused renal and toxicity markers’ leakage in the serum samples from PB-treated rats. Besides, the free radicals elicited by PB were the main factors for lowering the level of key antioxidant enzymes (SOD, CAT, and GR) along with GSH concomitant with an elevation in oxidative damage to lipids and proteins (MDA and carbonyl contents) in the target organ. Many cell-line- and animal-based studies have shown that the toxicant, PB, elicits pernicious effects in vivo mediated by the free radicals [21,32]. It is believed that the nano-sized NPs can easily get into the target cells and exert their effect on the structural, molecular, and functional domains of the cells depending on the extent of the dose and the period of exposure. On the other hand, the repeated dose of ZnO exerted a moderate level of nephrotoxicity, as all the parameters were altered mildly compared to the control. On the contrary, despite the reports on the toxicity of ZnO-NPs at a high dose [21], the present study aimed to harness the positive impact of the NPs at smaller doses. The NPs were found to be well tolerated by the animals as most of the studied parameters were close to the control levels. Intriguingly, the NPs were able to significantly ameliorate the PB-induced nephrotoxicity as evidenced by lowering the renal function and toxicity markers along with improvement in the activity of antioxidant enzymes and reduced glutathione. The NPs also protected from PB-induced MDA and carbonyl contents in the combination-treated groups. Because of the ceasing of PB-induced free radicals, the nuclear DNA of the target cells showed shorter tail-lengths, indicating their intactness as shown by the comet results. Further, the histological evaluation revealed a significant improvement in the microstructure of the renal tissue, confirming the biochemical and comet results. A similar protective efficacy of these NPs has been reported in many previously published reports [27,33,34]. Furthermore, we reported the ameliorative effect of the NPs against PB-induced hepatotoxicity in the rat model [35].

It is assumed that the NPs dissociate after getting into the target cells, leading to the leaching out of Zn^+2^ ions at a slow continuous rate. The ions can sensitize the cellular machinery, triggering the redox system in the cells. Zn, a co-enzyme of Cu-Zn SOD, triggers the whole antioxidant system in the cells affected by PB. Hence, an elevation in the activity of CAT and GR along with the replenishment of GSH was observed in the rats treated with the NPs. As the antioxidant enzymes started operating, the lipid peroxidation (MDA levels) and carbonyl content dropped in the same group significantly. Earlier, Bashandy et al. [26] also documented that ZnO NPs’ alleviative thioacetamide-induced toxicities in vivo.

It is noteworthy that Zn ions are immune boosters in nature. Hence, the NPs can orchestrate the immune system, heightening the pro-apoptotic factors (caspases, Bax) concomitant with stifling the anti-apoptotic proteins (BCL-2) in the PB-challenged cells. Some studies have shown that a suitable dose of NPs can dictate the PB-abused cells to undergo programmed cell death [34]. The present study also revealed that the proposed NPs can harmonize the cellular redox parameters and immune system, which is translated in the form of the attenuation of PB-induced toxic insults and cellular damage in the kidneys. These results are well supported by the histological analysis of renal tissues from PB-challenged groups treated with NPs. Their histology clearly showed that the NPs diminished the structural deformities and restored the microstructure of critical cellular organelles and overall cellular integrity. Further, these NPs have demonstrated their capability for the induction of autophagy along with apoptosis in some of the cell-line-based studies [36]. Many investigators have reported that free radicals elicited by the NPs target the nuclear DNA directly besides organelles such as mitochondria and lysosomes, leading to the induction of apoptosis, necrosis, or autophagy, subject to the existing cellular macroenvironment and microenvironment [33,36]. These NPs can access the mitochondria’s inner membrane and trigger the spill out of the pro-apoptosis proteins [37]. This consequently can lead to mitochondrial dysfunction or apoptosis induction. Further reports have indicated that the NPs can elevate LC3, which can induce autophagy and/or apoptosis. Studies have shown that the NPs enter the lysosome and trigger the gradual leaching out of Zn ^+2^ in an acidic environment outside the organelle [38,39]. Then, the active radicals can further invade the mitochondria, resulting in apoptosis or necrosis [40]. However, the NPs can trigger apoptosis by heightening autophagy by inhibiting PI3K/AKT/mTOR and halting their phosphorylation in vivo [41]. It was also highly speculated in the present investigation that the Zn ions can leach out from the NPs in the target cells’ heightened redox status, which can further facilitate apoptosis [34]. However, these speculations are subject to various factors including how the NPs act in a controlled fashion in prevailing redox status and if the extent of cellular damage and energy level is favorable for apoptosis [42,43].

In the present study, it is imminent that the consecutive doses of NPs administered accumulate in the target cells’ cytosol, mitochondria, and lysosomes. It can enhance cellular stress to a moderate level as the doses were repeated at particular gaps. Therefore, the present investigation entails that a moderate stress level maintained by a repeated low dose of the NPs at regular intervals favors the triggering of apoptosis and autophagy in vivo and in situ [6,42]. Furthermore, the NPs showed the resurrection of the mildly damaged cells evidenced by their improved biochemical parameters and histology. Besides, the release of Zn ions from the NPs is immunogenic, which can further facilitate cellular repair along with the stabilization of the nuclear DNA [44] in the target cells. These notions were evident from the result of the comet assay and biochemical analysis in this study. Nevertheless, further studies are warranted to disclose the exact mechanism involved in vivo.

## 4. Materials and Methods

### 4.1. Materials

The key chemicals and reagents used were bought from Sigma Aldrich (St. Louis, MO, USA) or EMD Millipore Merck (Darmstadt, Germany). The estimation kits used were purchased from either Linear or Span diagnostic kits (Spain).

### 4.2. Methods

#### 4.2.1. Animal Husbandry

Thirty-six Swiss albino male rats (100 ± 30 g, 7–8 weeks old) were procured from the Animal House of the Department of Zoology (KSU, Riyadh, Saudi Arabia). They were placed in a specially assigned treatment room in the Departmental Animal House (Department of Zoology, KSU, Riyadh). The room was equipped with all animal handling requirements and care with regular rat-feed and fresh tap water ad libitum. All the animals were kept for 10 days for acclimatization before starting the treatment. Finally, the rats were separated into six treatment groups (*n* = 6) as follows:

Group I: Control treated with saline only;
Group II: A single dose of KBrO_3_ at 100 mg/kg body weight [44,45];
Group III: ZnO at a dose of 5 mg/kg body weight twice a week for a month;
Group IV: ZnO-NP at a dose of 5 mg/kg body weight twice a week for a month;
Group V: A single dose of KBrO_3_ at a dose of 100 mg/kg + 5 mg/kg of ZnO administered twice a week for a month;
Group VI: A single dose of KBrO_3_ at a dose of 100 mg/kg + 5 mg/kg of ZnO-NP administered twice a week for a month.

After completion of the treatment, all the animals were sacrificed on the same day. All the animal handling procedures were conducted as per the ethics of animal experimentation (KSU Ethics Committee, Riyadh, Saudi Arabia).

#### 4.2.2. Preparation of Nanoparticles

The nanoparticles used in this study were prepared with green synthesis methods using the leaves of the Ochradenus arabicus (OA) plant. All the methods employed for synthesizing and characterizing the NPs were conducted as previously reported [45].

#### 4.2.3. Preparation of Biological Samples

After sacrifice, the primary target organ, the kidney, was washed with chilled phosphate-buffered saline. The samples were homogenized in Tris–KCl buffer (pH 7.4), and their supernatant was stored in the labeled vials at −80 °C after centrifugation (Eppendorf, Hamburg, Germany) till further analysis. Besides, the blood was also collected in vacuum tubes (BD Science, San Jose, CA, USA), which were centrifuged (Eppendorf, Germany) to retrieve the serum samples at 1200× *g* and were stored at −25 °C (Bosch, Stuttgart, Germany).

#### 4.2.4. Assessment of Kidney Function Markers

In the present study, urea, creatinine, and blood urea nitrogen (BUN) were chosen for the assessment of the functionality of the target organ. Commercial kits were used to measure all the parameters, either by Linear diagnostic kits (Amposta, Spain) and kits (Quimica Clinica Aplicada S. A., Amposta, Spain), following the manufacturer’s instructions (Catalogue Nos. 994997 and 998891).

#### 4.2.5. Estimation of Toxicity Burden on the Kidney

In the present investigation, serum albumin and glutamyl S-transferase (GST) were the parameters for assessing the target organ’s toxic burden. According to the concerned manual, they were estimated in the serum samples by the commercial kits (Quimica Clinica Aplicada S. A., Amposta, Spain, with Catalogue Nos. 997258 and 993561).

#### 4.2.6. Activity Assay of Antioxidant Enzymes

Superoxide dismutase (SOD), catalase (CAT), and glutathione reductase (GR) were chosen as the antioxidant enzymes. Their activity was measured by the established protocols [46,47,48], respectively (Supplementary File S1).

#### 4.2.7. Estimation of Reduced Glutathione Level

Reduced glutathione (GSH) is one of the significant cellular reductants in maintaining normal cellular redox status. It was also quantified by the method of [49] (Supplementary File S1).

#### 4.2.8. Assessment of Macromolecular Oxidative Damage

The damage to lipids and proteins by oxidative stress was measured by malondialdehyde (MDA) and carbonyl content level by the established protocols [50,51], respectively (Supplementary File S1).

#### 4.2.9. Comet Assay

This assay to measure nuclear DNA damage in the kidney cells was executed per the protocol of Singh et al. [52] with a few modifications [42] in the present investigation. The modification included the preparation of single cell suspensions of the target organ (0.5 g) in RPMI 1640 (2 mL) (Supplementary File S1).

#### 4.2.10. Histopathological Evaluation

The kidney tissue samples of the rats were fixed in 8% formalin and further processed, as mentioned in our previous studies [35]. The stained slides were evaluated by a light microscope (Leica, Wetzlar, Germany) blindfolded, and their photomicrographs were snapped by a high-definition digital microscopic camera (Leica MC 170 HD, Singapore).

#### 4.2.11. Statistical Analysis

All the data are depicted as the mean ± the SD and were analyzed by one-way ANOVA with Tukey’s post hoc multiple comparison test choosing a *p*-value ≤ 0.05 as statistically significant using the GraphPad Prism 5 software. *, **, and *** indicate statistically significant from the control (Group I) at *p* ≤ 0.05, *p* ≤ 0.01, and *p* ≤ 0.001, respectively, while #, ##, and ### indicate statistically significant from Group II at *p* ≤ 0.05, *p* ≤ 0.01, and *p* ≤ 0.001, respectively (*n* = 5–6).

## 5. Conclusions

The present study revealed the substantial ameliorative effect of ZnO-NPs against PB-induced nephrotoxicity in vivo. Hence, the NPs have great potential to be used along with medicines having nephrotoxicity as a major side effect and also to improve PB-based consumer items and industrial products.

## Figures and Tables

**Table 1:** Histological alteration in the tissue section of the kidney samples from: Group I (control); Group II (single dose of PB at 100 mg/kg); Group III (multiple doses of ZnO at 5 mg/kg); Group IV (multiple doses of ZnO-NP at 5 mg/kg); Group V (single dose of PB at 100 mg/kg with multiple doses of ZnO at 5 mg/kg; Group VI (single dose of PB at 100 mg/kg with multiple doses of ZnO-NPs at 5 mg/kg), where: “–“ indicates a lack of any structural change; “+” indicates a slight structural change; “++” indicates a moderate structural change; “+++” indicates a severe structural change when compared to the control.

Treatment Groups	I	II	III	IV	V	VI
Oedematous glomeruli	−	+++	++	−	+++	−
Hemorrhage	−	++	++	−	−	−
Infiltration of inflammatory cells	−	+++	++	+	++	+
Hyaline casts	−	++	++	−	++	+
Disintegrated nucleus	−	+	−	−	−	−

## Data Availability

All the data relevant to the work are included in the manuscript.

## Supplementary Materials

The following supporting information can be downloaded at: https://www.mdpi.com/article/10.3390/molecules28135084/s1, File S1: Methods. References [53,54,55] are cited in the supplementary materials.
