# Supplementary material for: Zinc Oxide Nanoparticles Blunt Potassium-Bromate-Induced Renal Toxicity by Reinforcing the Redox System
Source: Molecules. 2023 Jun 29;28(13):5084. doi: 10.3390/molecules28135084 (PMC10343659; doi:10.3390/molecules28135084)
Supplement: Supplementary file 1 [file molecules-28-05084-s001.zip › molecules-2441540-supplementary.pdf]

Assay of SOD

Superoxide dismutase activity was measured with the standard protocol (Marklund and Marklund, 1974 [8]). The enzymatic activity of Cu–Zn superoxide dismutase(Cu–ZnSOD) was assessed by auto-oxidation of pyrogallol in the presence of 100 mL of sample in succinate buffer (pH 7.36). Per minute change in OD at 412 nm by UV–visible spectrophotometer ((Pharmacia Biotech, England).) was recorded for calculation.

Assay of CAT

Catalase (CAT) was assayed by measuring the inhibition of decomposition of hydrogen peroxide by 50 mL of sample in potassium–phosphate buffer (pH 7.0) was read at 240 nm per minute by UV–visible spectrophotometer (Aebi, 1984 [1]). Their specific values were expressed in units per milligram of protein in the sample.

Assay of GST

Glutathione-S-transferase (GST) was assayed by following the method of Habig et al. (1974) [3]. 100 mL of sample was allowed to react with 2.7mL of reduced glutathione (30.73 mg% in potassium–phosphate buffer at pH 6.5) and 200 mL chloro-dinitrobenzene (1mM in acetone). After vortex mixing, the reading was taken at 340 nm per minute against distilled water (blank) on the spectrophotometer.

Assay of GSH

The measurement of reduced glutathione (GSH) was conducted by method of Jollow et al. (1974) [6]. 0.5 mL of tissue homogenate was allowed to incubate with 0.5 mL of 5-sulfosalicylic acid (4 g%) for 1h at 4°C followed by its centrifugation at 1200×g in cold. The supernatant (0.2mL) was mixed with 0.2mL of Ellman's reagent (4mg/mL in potassium–phosphate buffer of pH 7.4) in the same buffer (1.1 mL). The reading was taken at 412 nm against the buffer having Ellman's reagent. The level of GSH was reported in nmoles per milligram of protein in the sample.

Assay of lipid peroxidation

The extent of lipid peroxidation was measured in terms of total malondialdehyde (MDA) level by the protocol of Beuge and Aust (1978) [2]. According to the protocol, 0.5mL of the tissue homogenate was boiled with 0.5mL of thiobarbituric acid (0.67g %) and 0.5mL of trichloroacetic acid (30g %) in falcon centrifuge tubes for 30 min. They were then centrifuged at 2000×g for 10 min and their pink supernatant was read at 530 nm by the spectrophotometer (Pharmacia Biotech, England).

Assay of protein oxidation (carbonyl content)

The total carbonyl content assay was conducted by mixing 1mL of sample with 0.4mL of trichloroacetic acid (40g%) and centrifuged (Eppendorff, England) at 3000×g for 10min in cold (Levine et al., 1994 [7]). After that the pellet was washed with phosphate buffer saline (100mM, pH 7.4) and allowed to incubate with 0.4mL of 2,4-dinitrophenylhydrazine (200mM prepared in 4N HCl) at 37◦C for 1h in water bath incubator shaker (Eppendorf, Germany). After that, they were centrifuged at 3000×g for 10min.The pellet was further centrifuged with 40g% trichloroacetic acid. The resulting pellet was mixed with ethanol and ethyl acetate mixture (1:1) followed by centrifugation. ,The pellet was incubated with 1mL of guanidine HCl (6M) after washing with phosphate-buffered saline twice. Finally, its absorbance was observed at 360 and 280nm and the total carbonyl content was reported in nanomoles per milligram of the tissue sample.

Comet Assay

The assay was executed in alkaline conditions following the protocol of Singh et al. (1988) [10] with few standardized modifications (Hassan et al., 2012 [4]). Fully frosted slides precoated with 1% NMA (as base layer) at 60˚C were prepared day prior to the sacrificing of the animals. Almost 10,000 cells were isolated from cell suspension of every organ (freshly prepared). They were mixed with 80 mL 1% of LMPA for preparation of the working cell suspension for sample. This suspension was then pipetted over the base layer at 37˚C followed by placing coverslips. After solidification of second layer in cold, the coverslips were removed and a third layer of 0.5% LMPA (80 mL) was pipetted over followed by putting coverslips over it. After solidification of the third layer, the coverslips were removed and the slides were immersed in cold lysing solution (2.5M

NaCl +100mM EDTA + 10mM tris-base + 1% triton X-100) with pH 10 for 3h. Then, it was subjected to unwinding for 30 min in alkaline electrophoretic running buffer (300 mM NaOH + 1mM EDTA) at pH 13 in coplin jars. After this, electrophoresis was started upto 35 min at 4°C with constant field strength of 0.74 volts/cm and current strength of 300 mA. The slides were washed with cold saline and subjected to neutralization by neutralizing buffer (0.4M tris-base) with pH 7.5 followed by washing with cold saline. This process of neutralization was repeated twice. The slides were then stained with 75 mL ethidium bromide (20 mg/mL) for 5min. Finally, the slides were rewashed with chilled saline and coverslips were placed on and finally, they were stored in humidified slide box at 4◦C till their analysis. The slides were scored for comet tail length with the help of CX41 fluorescent microscope (Leica, Germany) coupled with an image-analysis system (Komet 5.5, Kinetic imaging, Liverpool, U.K.) attached with integrated CC camera. The comets were scored at the magnification of 100× and images from 50 cells (25 from each replicate slide) were scored. Comet tail length (migration of DNA from its nucleus in mm) is the most established parameter to assess the nuclear DNA damage that was automatically generated by Komet 5.5image-analyzing system in numerical values for the slides (Naseem et al., 2015 [9]).

Histological analysis

The fresh tissue samples were processed by dipping in 8% formaldehyde solution in phosphate-buttered saline (PBS) after washing and dehydrating in a series of alcohol dilutions. They were then embedded in paraffin to prepare ~5-7 μm microtome sections staged on the slides stained with hematoxylin and eosin. The histopathological alteration in the sections was examined in a blindfolded manner using a light microscope (Leica DMRB/E Heerbrugg, Switzerland) with an attached HD camera (Leica MC 170 HD, Singapore). Photomicrographs of the sections were snapped, which were digitally upgraded using Adobe Photoshop (Adobe Systems, Mountain View, CA) (Hassan et al., 2019 [5]).

References:

1. Aebi H.Catalase in vitro. Meth Enzymol 1984;105:121–6.
2. Beuge JA,Aust SD. Microsomal lipidperoxidation. Meth Enzymol 1978;52:302–10.
3. Habig WH, Pabst MJ, Joko by WB.Glutathione S-transferase:thefirstenzymatic step in mercapturic acid formation.JBiolChem1974;249(22):7130–9.
4. Hassan I,Chibber S,Khan AA,Naseem I. Riboflavin ameliorates cisplatin induced toxicities under photoillumination. PlosOne 2012;7(5):e36273.

5. Hassan, I., Husain, F.M., Khan, R.A. *et al.* Ameliorative effect of zinc oxide nanoparticles against potassium bromate-mediated toxicity in Swiss albino rats. *Environ Sci Pollut Res* **26**, 9966–9980 (2019).
6. Jollow DJ, Mitchell JR,Zampaglione N,Gillette JR. Bromobenzene induced liver necrosis: protective role of glutathione and evidence for 3,4-bromobenzene oxide as hepatotoxic metabolite.Pharmacology 1974;11:151–69.
7. Levine RL,Williams J,Stadman ER,Shacter E. Carbonyl assay for determination of oxidatively modified proteins.Meth Enzymol 1994;233:346–57.
8. Marklund S, Marklund G. Involvement of superoxide anion radical in the auto- oxidation of pyrogallol and a convenient assay for superoxide dismutase. Eur J Biochem 1974;47:469–74.
9. Naseem I, Hassan I, Alhazza IM, Chibber S. Protective effect of riboflavin on cisplatin induced toxicities: a gender-dependent study. J Trace Elem Med Biol. 2015 Jan;29:303-14. doi: 10.1016/j.jtemb.2014.08.003. Epub 2014 Aug 20. PMID: 25242266.
10. Singh NP,McCoy MT,Tice RR,Schneider EL.Asimple technique for quantification of low levels of DNA damage in individual cells.Exp Cell Res 1988;175:184–91.
